# Sedative-sparing effect of acupuncture in gastrointestinal endoscopy: systematic review and meta-analysis

**DOI:** 10.3389/fmed.2023.1189429

**Published:** 2023-06-16

**Authors:** Yun Yang, Haiyang Ji, Yunqiong Lu, Jue Hong, Guang Yang, Xiehe Kong, Jie Liu, Xiaopeng Ma

**Affiliations:** ^1^Yueyang Hospital of Integrated Traditional Chinese and Western Medicine, Shanghai University of Traditional Chinese Medicine, Shanghai, China; ^2^Shanghai Research Institute of Acupuncture and Meridian, Shanghai University of Traditional Chinese Medicine, Shanghai, China

**Keywords:** acupuncture, sedation, propofol, gastrointestinal endoscopy, colonoscopy, gastroscopy, wake-up time, adverse event

## Abstract

**Objective:**

This study aimed to perform a systematic review and meta-analysis to identify the efficacy of acupuncture therapy (including manual acupuncture and electroacupuncture) performed before or during gastrointestinal endoscopy with propofol as the main sedative, compared with placebo, sham acupuncture, or no additional treatment other than the same sedation.

**Methods:**

A systematic search was performed through PubMed, Embase, Web of Science, Cochrane Library, Chinese Biomedical Databases (CBM), Wanfang database, China National Knowledge Infrastructure (CNKI), SinoMed, and Chinese Scientific Journal Database (VIP) to collect randomized controlled trials published before 5 November 2022. Bias assessment of the included RCTs was performed according to Version 2 of the Cochrane risk-of-bias tool for randomized trials (RoB 2). Stata16.0 software was used to perform statistical analysis, sensitivity analysis, and publication bias analysis. The primary outcome was sedative consumption, and the secondary outcomes included the incidence of adverse events and wake-up time.

**Results:**

A total of 10 studies with 1331 participants were included. The results showed that sedative consumption [mean difference (MD) = −29.32, 95% CI (−36.13, −22.50), *P* < 0.001], wake-up time [MD = −3.87, 95% CI (−5.43, −2.31), *P* < 0.001] and the incidence of adverse events including hypotension, nausea and vomiting, and coughing (*P* < 0.05) were significantly lower in the intervention group than in the control group.

**Conclusion:**

Acupuncture combined with sedation reduces sedative consumption and wake-up time compared with sedation alone in gastrointestinal endoscopy; this combined approach allows patients to regain consciousness more quickly after examination and lower the risk of adverse effects. However, with the limited quantity and quality of relevant clinical studies, caution must be applied until more high-quality clinical studies verify and refine the conclusions.

**Systematic review registration:**

https://www.crd.york.ac.uk/prospero/display_record.php?, identifier: CRD42022370422.

## 1. Introduction

Gastrointestinal endoscopy is essential in diagnosing, managing, and treating inflammatory bowel disease and other diseases (1). The wide application of sedation in gastrointestinal endoscopy reduces anxiety and discomfort during endoscopy, increases patient tolerance and satisfaction, facilitates clinical operation and treatment, and improves examination quality (2). However, adverse reactions such as hypotension and respiratory depression often occur during sedation with propofol (3), a commonly used sedative in gastrointestinal endoscopy with its application in gastroscopy and colonoscopy both over 60% as a study of 2,758 hospitals in China showed (4). In addition, sedatives may also lead to cognitive impairment after endoscopy (5) and a higher overall risk of perforation, bleeding, and other complications (6).

Acupuncture is a promising technique for relieving patient's anxiety before an operation, reducing sedative consumption, postoperative pain, and adverse events, promoting the functional recovery of patients (7), as well as cutting the cost caused by sedative usage (8), which has shed new light on the research of gastrointestinal endoscopy techniques such as painless colonoscopy (9). As less attention was paid to the effects of acupuncture on sedation in gastrointestinal endoscopy in prior systematic reviews and meta-analyses (10–12), and the number of included studies was limited, here we aimed to perform an analysis to identify the efficacy of acupuncture therapy (including manual acupuncture and electroacupuncture) performed before or during gastrointestinal endoscopy with propofol as the main sedative, compared with placebo, sham acupuncture, or no additional treatment other than the same sedation.

## 2. Methods

### 2.1. Protocol and registration

The protocol for this systematic review and meta-analysis followed the Preferred Reporting Items for Systematic Review and Meta-Analysis Protocols checklist. This study has been registered on the PROSPERO platform [CRD42022370422].

### 2.2. Search strategy

A systematic search was performed through PubMed, Embase, Web of Science, Cochrane Library, Chinese Biomedical Databases (CBM), Wanfang database, China National Knowledge Infrastructure (CNKI), SinoMed, and Chinese Scientific Journal Database (VIP) from their inception to 5 November 2022 (Table 1). The original languages of the retrieved articles were limited to English and Chinese, and the involved references were searched manually.

**Table 1:** Details of search strategy in PubMed.

	**Query**
#1	(acupuncture therapy[MeSH Terms]) OR (acupuncture[MeSH Terms]) OR (manual acupuncture[Title/Abstract]) OR (electroacupuncture[Title/Abstract]) OR (auricular acupuncture[Title/Abstract]) OR (scalp acupuncture[Title/Abstract]) OR (needling[Title/Abstract]) OR (acupoint[Title/Abstract]) OR (acupuncture point[Title/Abstract])
#2	(endoscopy, gastrointestinal[MeSH Terms]) OR (gastroscopy[Title/Abstract]) OR (colonoscopy[Title/Abstract]) OR (balloon enteroscopy[Title/Abstract]) OR (duodenoscopy[Title/Abstract]) OR (esophagoscopy[Title/Abstract]) OR (proctoscopy[Title/Abstract]) OR (capsule endoscopy[Title/Abstract])
#3	(analgesia and anesthesia[MeSH Terms]) OR analgesia[Title/Abstract] OR anesthesia[Title/Abstract] OR sedation[Title/Abstract]
#4	clinical[Title/Abstract] OR trial[Title/Abstract] OR randomized[Title/Abstract] OR controlled[Title/Abstract]
#5	#1 AND #2 AND #3 AND #4

### 2.3. Eligibility criteria

#### 2.3.1. Inclusion criteria

The inclusion criteria were as follows: (1) participants: patients ≥ 18 years old who met the requirements of gastrointestinal endoscopy; (2) interventions: manual acupuncture or electroacupuncture (without limiting the acupuncture points, manipulations, or depth) performed before or during gastrointestinal endoscopy with propofol as the main sedative; (3) control: placebo, sham acupuncture, or no additional treatment, with the same sedative (s) as in the intervention group; (4) outcomes: sedative consumption as the primary outcome, and the incidence of adverse events (defined as respiratory depression/bradycardia/hypotension/hypoxemia/nausea or vomiting/abdominal distension/dizziness/coughing/restlessness during or after gastrointestinal endoscopy) and wake-up time (from the end of the examination to the time when patients can correctly answer basic questions) as the secondary outcomes; (5) study design: randomized controlled trials (RCTs).

#### 2.3.2. Exclusion criteria

Exclusion criteria were as follows: (1) participants: patients with obvious contraindications for gastrointestinal endoscopy or a history of gastrointestinal resection; (2) interventions/control: report neither the disappearance of eyelash reflex nor losing response after calling; (3) study design: case reports, reviews, study protocols, or conference papers; (4) the same studies repeatedly published (only the earliest published one was included); (5) studies with unpublished, unavailable or incomplete original data.

### 2.4. Study selection and data extraction

Two reviewers independently applied eligibility criteria and selected studies for inclusion in this review. After duplicates were removed, they screened the titles and abstracts for all the records and then assessed full-text articles of the remaining records for eligibility. Any disagreements during the selection process were resolved through discussions with a third reviewer.

The following data were extracted from the studies, including study title, first author, year of publication, sample size, baseline characteristics (gender and age) of participants, type of gastrointestinal endoscopy, sedatives with their usage and dosage, acupuncture measures in the intervention group (types of acupuncture, stimulation method, and acupoints), and outcomes (sedative consumption, the incidence of adverse events, and wake-up time). Two reviewers independently extracted the study data, and any disagreements during the extraction process were settled through discussions with a third reviewer. The authors were contacted for any missing data. An Excel spreadsheet was used to record the data.

### 2.5. Risk of bias assessment

Bias assessment of the included RCTs was performed independently by two reviewers according to Version 2 of the Cochrane risk-of-bias tool for randomized trials (RoB 2), and any disagreements during the extraction process were resolved through discussions with a third reviewer. The domains of bias assessment were as follows: risk of bias arising from the randomization process, risk of bias due to deviations from the intended interventions (effect of assignment to intervention, or effect of adhering to intervention), risk of bias due to missing outcome data, risk of bias in the measurement of the outcome, risk of bias in the selection of the reported result. Finally, an overall risk of bias was generated. The risk of bias was described as a low risk of bias, some concerns, or a high risk of bias.

### 2.6. Statistical analysis

Continuous data were expressed as mean difference (MD) and 95% confidence interval (CI), and dichotomous data as risk ratio (RR) and 95% CI. *P* < 0.05 indicated that the difference was statistically significant. Due to the heterogeneity caused by the diversity of acupuncture treatment methods (differences in stimulation methods, acupoint selection, depth of insertion, stimulation time, etc.) in acupuncture-related studies, a random effects model was used, and the source of heterogeneity was analyzed through subgroup analysis and sensitivity analysis. Stata16.0 software was used to perform statistical analysis. Sensitivity analysis was used to assess the robustness of the results. Subgroup analysis was used to determine whether the summary effects varied in relation to pre-specified clinical characteristics of the trials included, such as the type of gastrointestinal endoscopy (gastroscopy/colonoscopy) and the number of kinds of sedatives used. Publication bias was evaluated by Egger's test.

## 3. Results

### 3.1. Search results

**Figure 1:**
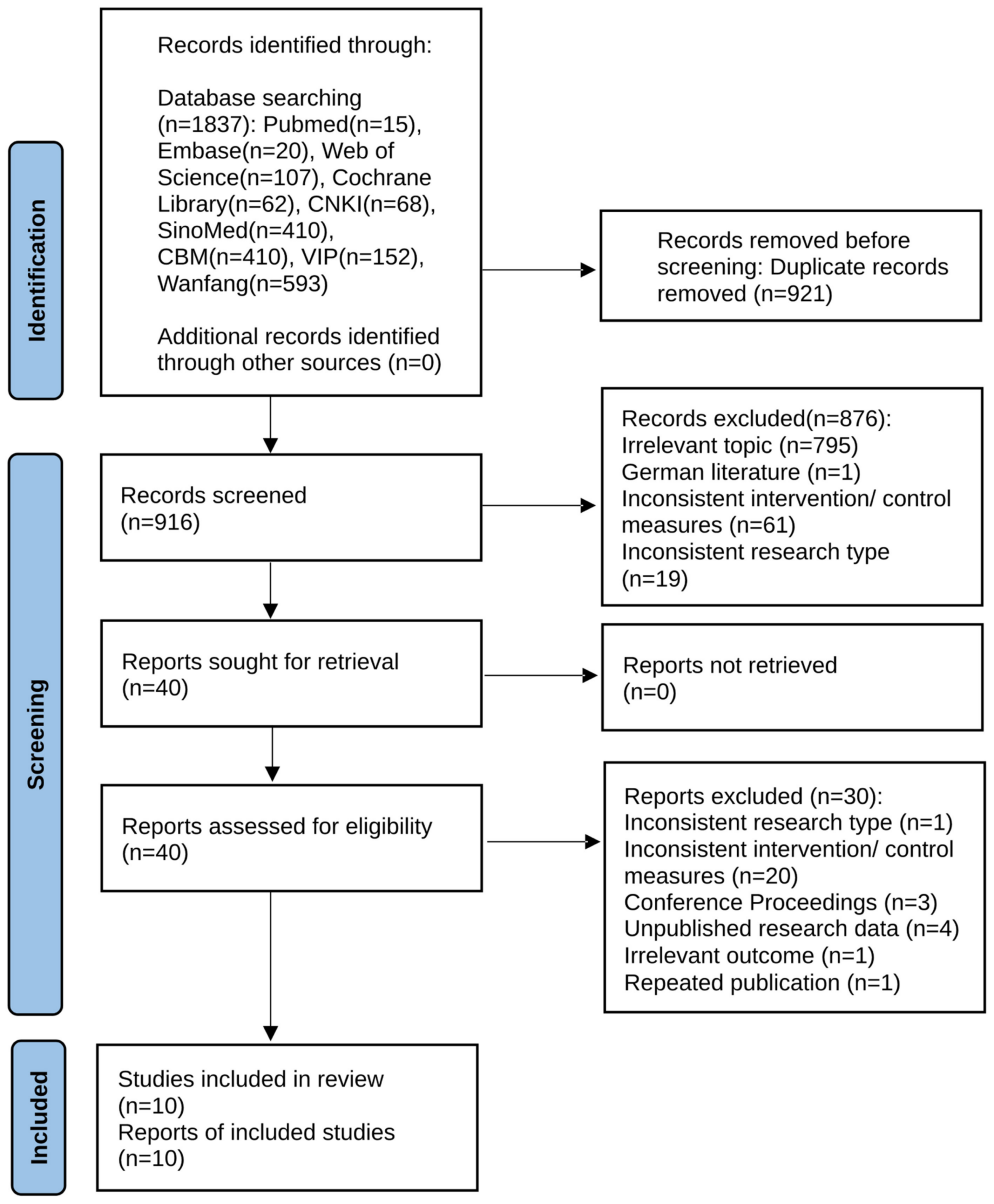
PRISMA flow diagram of article screening. This is a PRISMA 2020 flow diagram for new systematic reviews which included searches of databases and registers only.

Our systematic search identified 1,837 articles of interest, among which 916 articles remained after Endnote automatic and manual duplicate checking. After excluding 876 articles through the screening of titles and abstracts, full-text screening of the remaining 40 articles was performed, resulting in the selection of 10 articles for inclusion in the meta-analysis with 1,331 participants, including one English article and nine Chinese articles (Figure 1).

### 3.2. Characteristics of the included studies

The characteristics of the 10 RCTs included in the meta-analysis are summarized in Table 2.

**Table 2:** Basic characteristics of included articles.

**References**	**Sample size (I/C, *n*)**	**Baseline characteristics**		**Type of endoscopy**	**Sedatives in the intervention group and the control group**		**Types of acupuncture in the intervention group**	**Outcomes**
		**Gender (M/F**, ***n*****)**	**Age (I/C)**		**For induction of sedation**	**For maintenance of sedation**		
He et al. (13)	90/91	86/95	49.34 ± 1.11/49.73 ± 1.12	Colonoscopy	Nalbuphine (0.025 mg/kg) and Propofol (0.5 mg/kg)	Propofol	MA (wrist-ankle Acupuncture)	
Zhu (14)	80/80	88/72	49.16 ± 10.18/48.57 ± 11.03	Colonoscopy	Propofol (1 mg/kg)	Propofol	MA and EA	
Dong et al. (15)	60/60	67/53	76.54 ± 6.19/76.82 ± 7.00	Gastroscopy	Nalbuphine (0.1 mg/kg) and Propofol (2.5 μg/ml)	Propofol	MA (wrist-ankle acupuncture)	
Luo et al. (16)	60/60	67/53	53.5 ± 9.6/52.8 ± 9.7	Colonoscopy	Propofol (1 mg/kg)	Propofol	MA	
Luo et al. (9)	40/40	42/38	48.5 ± 10.5/48.8 ± 10.6	Colonoscopy	Propofol (1–2 mg/kg)	Propofol	MA	
Zheng et al. (17)	40/40	43/37	53.7 ± 12.7/50.7 ± 12.1	Colonoscopy	Propofol (1.5–2.0 mg/kg)	Propofol	EA	
Luo et al. (18)	60/60	69/51	43.8 ± 11.0/44.0 ± 10.8	Colonoscopy	Propofol (1–2 mg/kg)	Propofol	MA	
Wu et al. (19)	70/70	82/58	35–70	Gastroscopy	Fentanyl (1.0 μg/kg) and propofol (1.5–2.0 mg/kg)	Propofol	EA	
Chen et al. (20)	45/45	45/45	40.89 ± 13.88/41.44 ± 15.53	Gastroscopy	Propofol (1.5 mg/kg)	Propofol	EA	
Chen et al. (21)	120/120	138/102	25–70	Gastroscopy	Propofol (2 mg/kg)	Propofol	EA	

I, intervention group; C, control group; MA, manual acupuncture; EA, electroacupuncture; , sedative consumption; , wake-up time; , the incidence of adverse events.

### 3.3. Risk of bias assessment of the included studies

**Figure 2:**
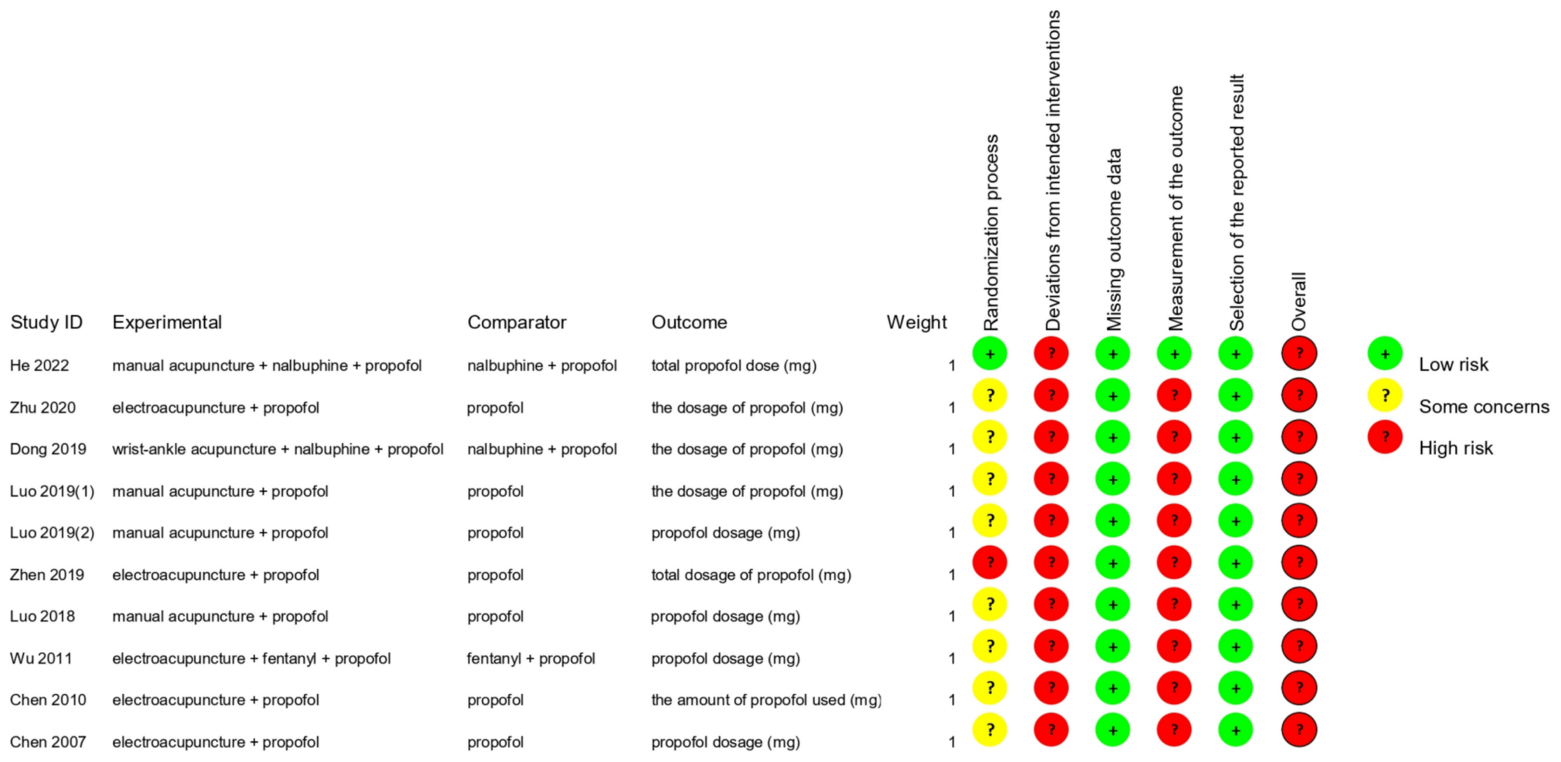
Risk of bias of each included study. Bias assessment of each included RCTs was performed according to RoB 2.

**Figure 3:**
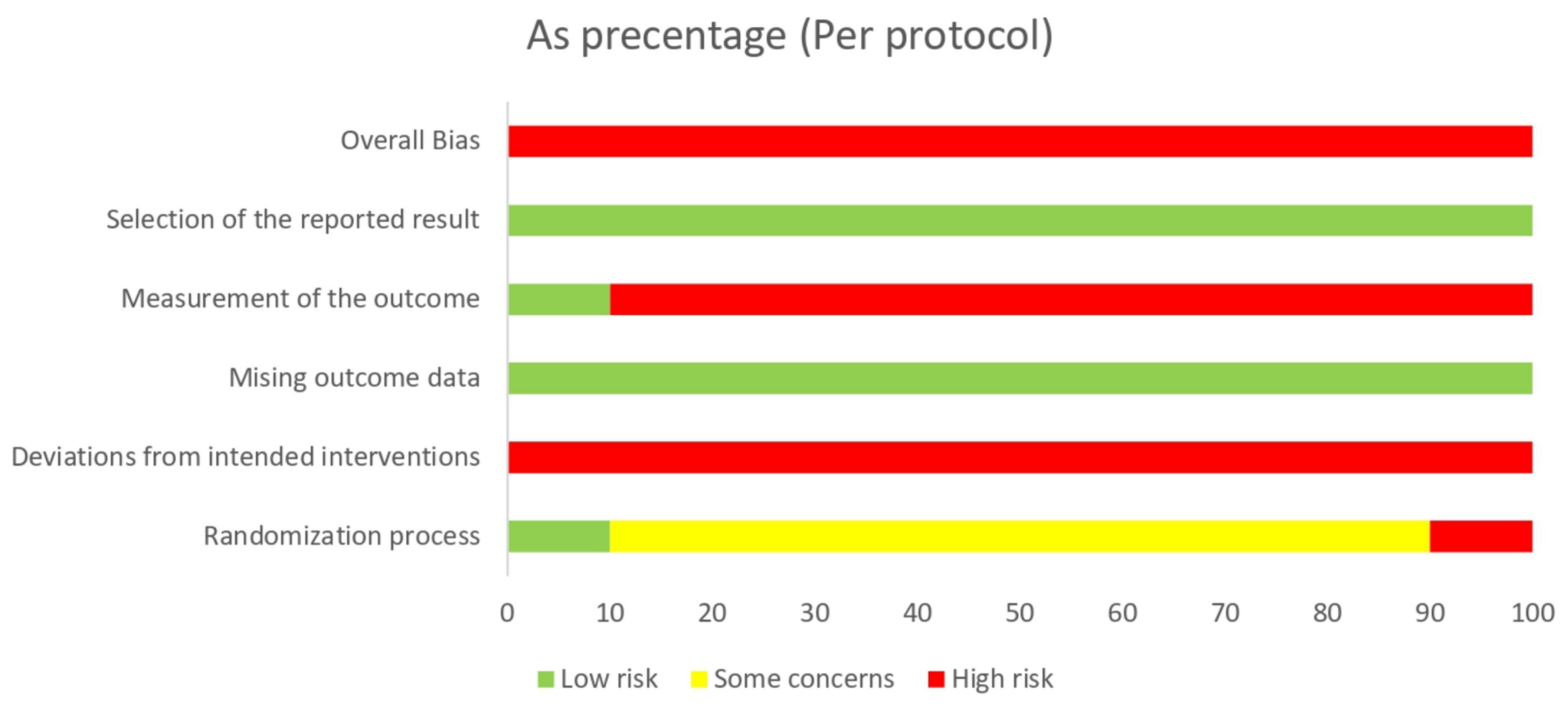
Summary of risk of bias of the included studies. Summary of bias assessment of included RCTs was performed according to RoB 2.

In the assessment of domain 1 (randomization process), only one study (13) was evaluated as having a low risk of bias, in which the random component used in the allocation sequence generation process was specified, and the allocation sequence was concealed until participants were enrolled and assigned to interventions. The rest of the trials failed to ensure the allocation sequences were both random and concealed. Eight trials (9, 14–16, 18–21) failed to ensure that the allocation sequences were random and concealed and thus rated as existing concerns. One study (17) where the patients were grouped according to their willingness was rated high risk of bias. In domain 2 (deviations from intended interventions), whether an appropriate analysis was used to estimate the effect of adhering to the intervention remained unknown, and therefore all studies were rated as having a high risk of bias. For the assessment of domain 3 (missing outcome data) and domain 5 (selection of the reported result), the 10 studies were all rated as having a low risk of bias. In domain 4 (measurement of the outcome), the outcome assessors in almost all the studies [except one (13)] were aware of the intervention received by study participants, resulting in the likelihood that the assessment of the outcome was influenced by the assessors' knowledge of intervention received. According to the signaling questions and assessment process of RoB 2.0 in each field, the 10 included studies were rated as having a high risk of bias in the overall bias assessment (Figures 2, 3; Table 3).

**Table 3:** Risk of bias of each included study.

**References**	**Randomization process**	**Deviations from intended interventions**	**Missing outcome data**	**Measurement of the outcome**	**Selection of the reported result**	**Overall bias**
He et al. (13)	Low	High	Low	Low	Low	High
Zhu (14)	Some concerns	High	Low	High	Low	High
Dong et al. (15)	Some concerns	High	Low	High	Low	High
Luo et al. (9)	Some concerns	High	Low	High	Low	High
Luo et al. (16)	Some concerns	High	Low	High	Low	High
Zheng et al. (17)	High	High	Low	High	Low	High
Luo et al. (18)	Some concerns	High	Low	High	Low	High
Wu et al. (19)	Some concerns	High	Low	High	Low	High
Chen et al. (20)	Some concerns	High	Low	High	Low	High
Chen et al. (21)	Some concerns	High	Low	High	Low	High

### 3.4. Results of meta-analysis

#### 3.4.1. Heterogeneity analysis of included studies

Meta-analysis results of propofol dosage (*I*^2^ = 97.8%) and wake-up time (*I*^2^ = 96.5%) presented significant heterogeneity, the possible sources of which were speculated as follows: The randomization scheme of the studies was imperfect. The sample size varied considerably across studies from 80 (9, 17) to 240 patients (21). The baselines of patients were not completely consistent among different studies. Differences in acupoints, stimulation intensity and frequency, and stimulation time of acupuncture might affect the experimental results to some extent (17, 22).

#### 3.4.2. Sedative (propofol) consumption

**Figure 4:**
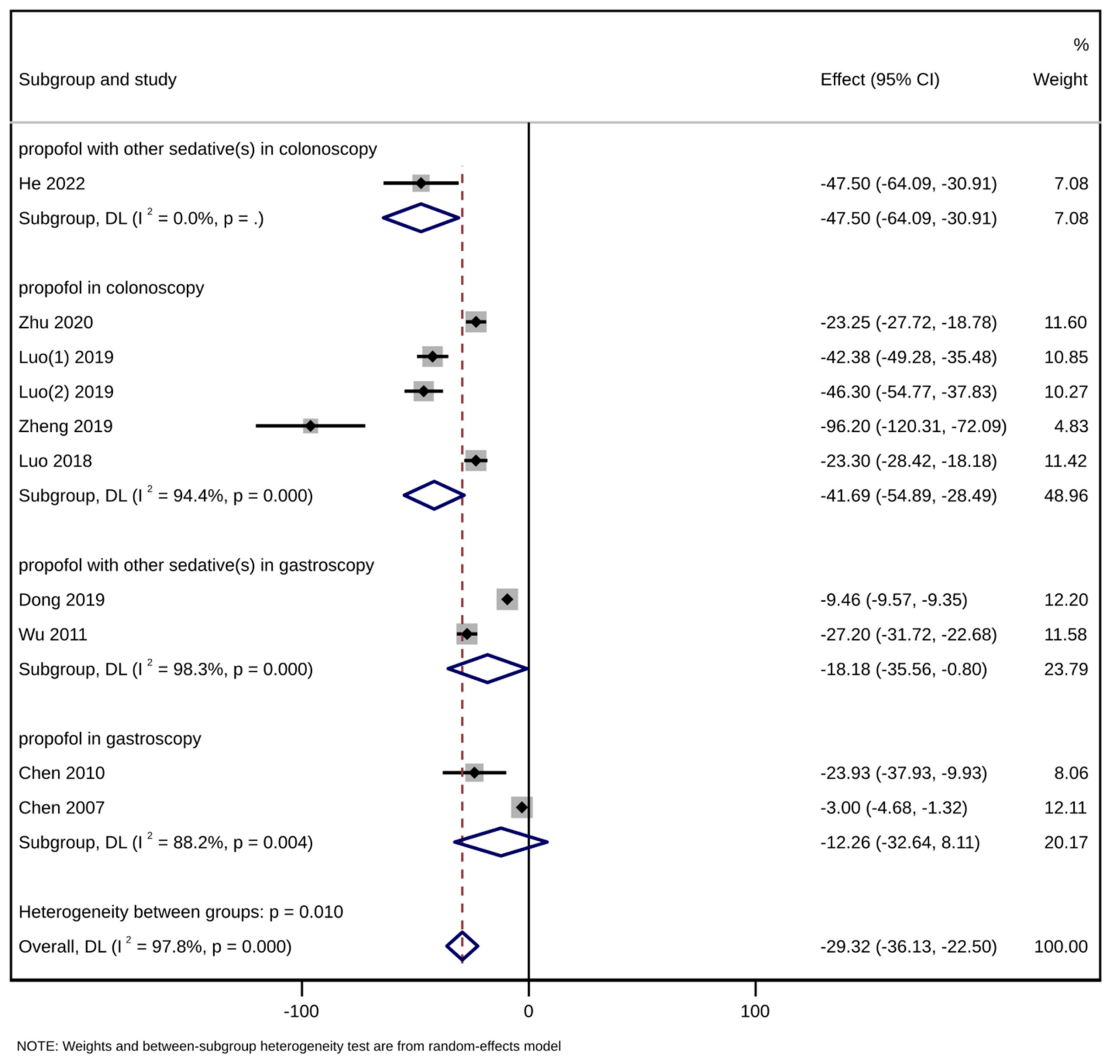
Forest plot of subgroup meta-analysis of sedative (propofol) consumption (mg). This is a meta-analysis of studies comparing acupuncture combined with sedative(s) vs. sedative(s) using the random-effects model with the effect showing the mean difference of sedative (propofol) consumption. A subgroup analysis has been conducted according to the type of gastrointestinal endoscopy (gastroscopy/colonoscopy) and the number of kinds of sedatives used. CI, confidence interval.

**Figure 5:**
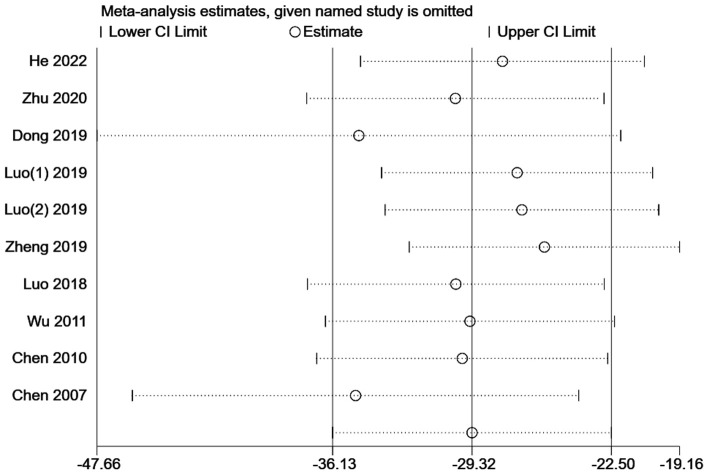
Leave-one-out sensitivity analysis of sedative (propofol) consumption for acupuncture combined with sedative(s) vs. sedative(s). CI, confidence interval.

**Figure 6:**
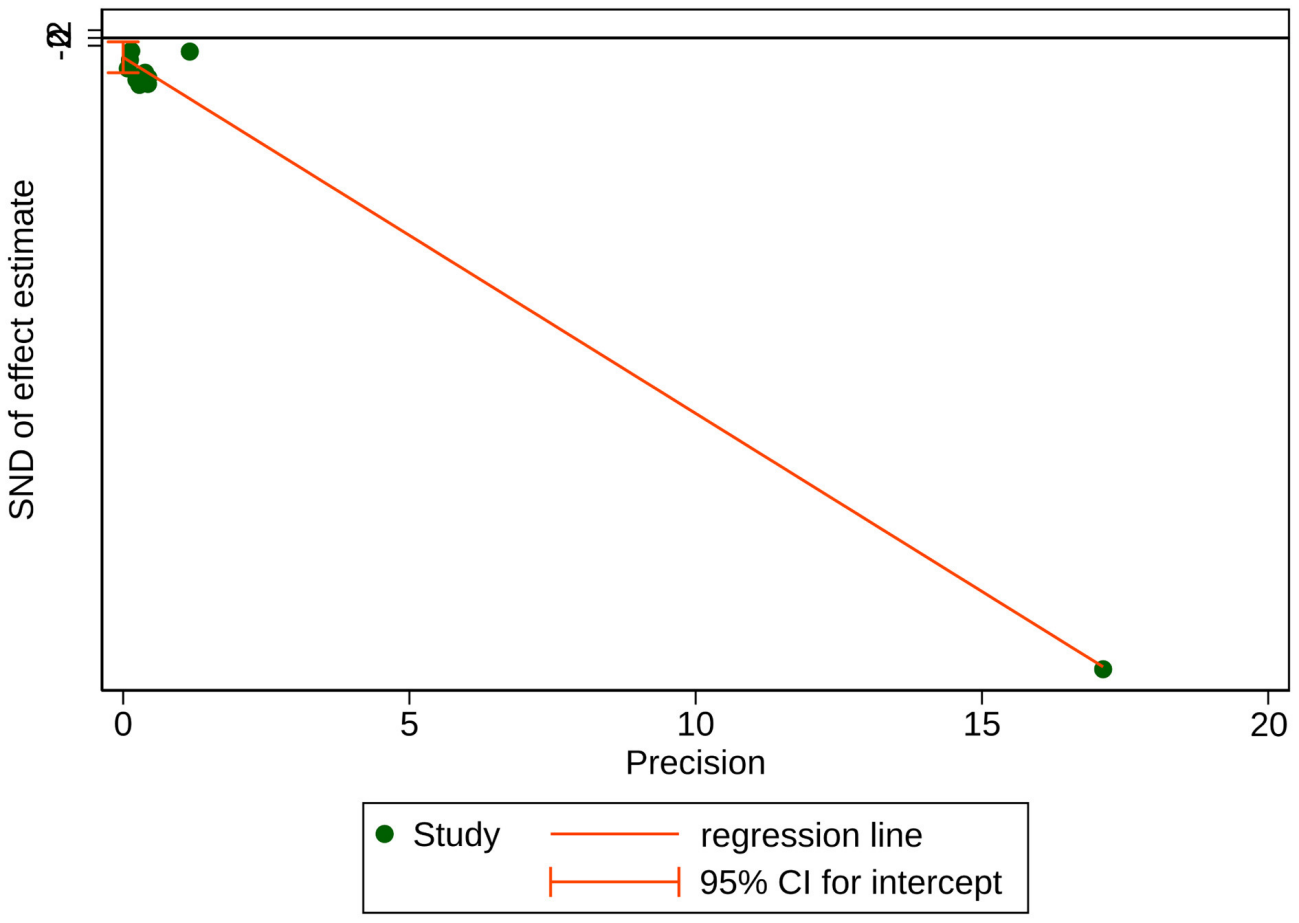
Egger's test for publication bias of sedative (propofol) consumption. Bias = −4.957401, Slope = −9.135269, *P*-value = 0.020 < 0.05.

The 10 articles all reported the consumption of the sedative propofol, with a total sample size of 1,331 participants [MD = −29.32, 95% CI (−36.13, −22.50), *P* < 0.001]. Due to high heterogeneity (*I*^2^ = 97.8%), a subgroup analysis was conducted according to the type of gastrointestinal endoscopy (gastroscopy/colonoscopy) and the number of kinds of sedatives used (Figure 4). The group concerning propofol used together with other sedative (s) in colonoscopy included only one study (13) [MD = −47.50, 95% CI (−64.09, −30.91), *P* < 0.001]. The group of propofol alone in colonoscopy included five studies (9, 14, 16–18) (MD = −41.69, 95% CI [(−54.89, −28.49), *P* < 0.001]. The group concerning propofol used together with other sedative (s) in gastroscopy included two studies (15, 19) [MD = −18.18, 95% CI (−35.56, −0.80), *P* = 0.040]. In those three subgroups, sedative (propofol) consumption was significantly lower in the intervention group than in the control group. However, when it comes to the group of propofol alone in gastroscopy containing two studies (20, 21), the difference in sedative (propofol) consumption was not statistically significant [MD = −12.26, 95% CI (−32.64, 8.11), *P* = 0.238]. In addition to subgroup analysis, a univariate meta-regression analysis concerning non-methodological factors including publication year (*P* = 0.170), sample size (*P* = 0.125), and female ratio (*P* = 0.280) of the included studies found no statistically significant results, leaving the high heterogeneity unsolved. Although a subsequent sensitivity analysis suggested stable results (Figure 5), Egger's test suggested a significant publication bias (*P* = 0.020 < 0.05) (Figure 6), and the conclusion that acupuncture reduced sedative consumption in gastrointestinal endoscopy is questionable.

#### 3.4.3. Wake-up time

**Figure 7:**
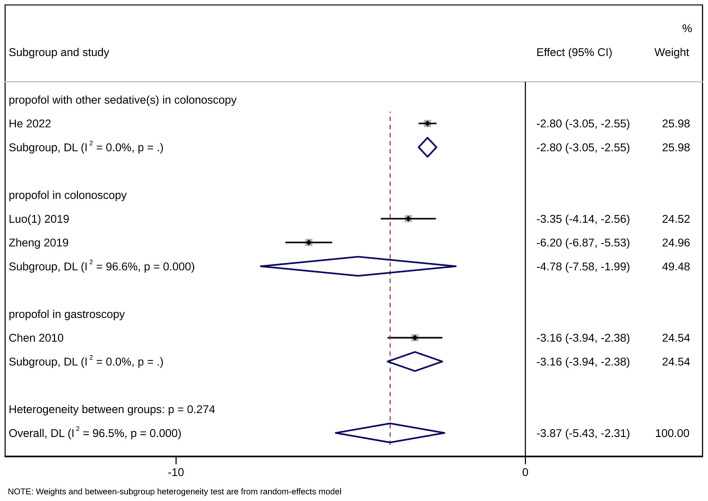
Forest plot of subgroup meta-analysis of wake-up time. This is a meta-analysis of studies comparing acupuncture combined with sedative(s) vs. sedative(s) using the random-effects model with the effect showing the mean difference of wake-up time. A subgroup analysis has been conducted according to the type of gastrointestinal endoscopy (gastroscopy/colonoscopy) and the number of kinds of sedatives used. CI, confidence interval.

**Figure 8:**
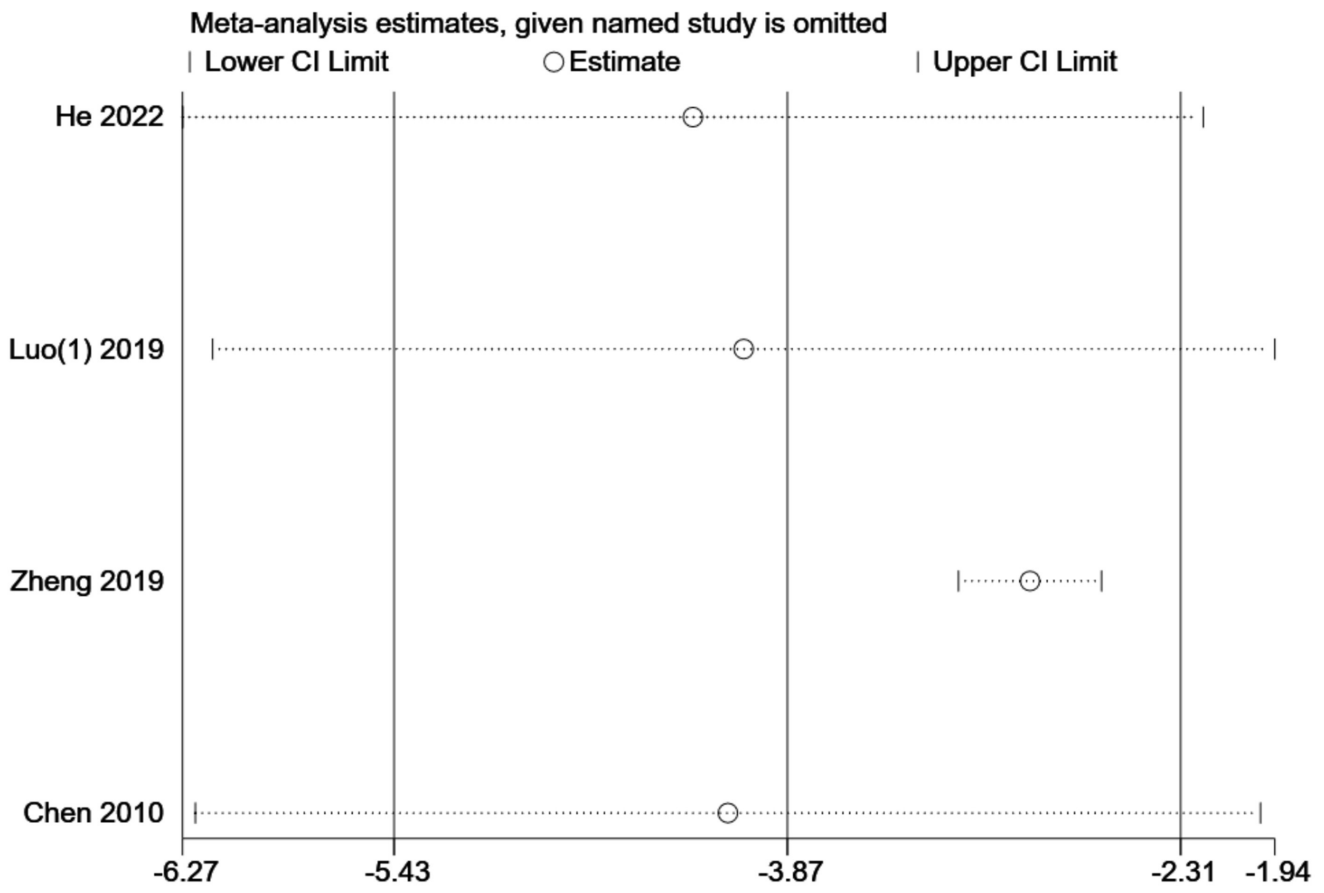
Leave-one-out sensitivity analysis of wake-up time for acupuncture combined with sedative(s) vs. sedative(s). CI, confidence interval.

**Figure 9:**
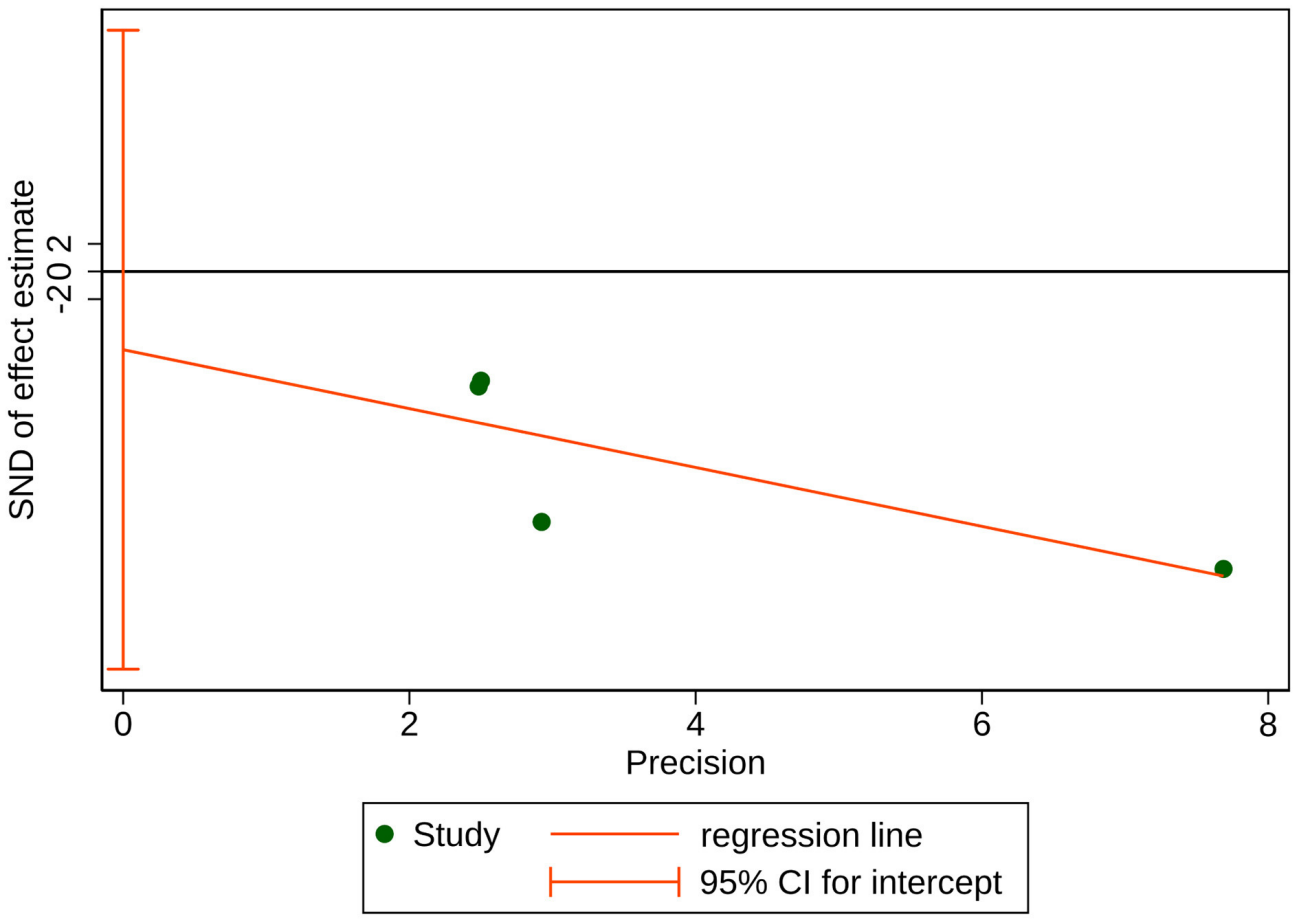
Egger's test for publication bias of wake-up time. Bias = −5.659218, Slope = −2.131156, *P*-value = 0.403 > 0.05.

Four studies (13, 16, 17, 20) with a total sample size of 471 patients reported wake-up time in the outcome indicators. The results showed that acupuncture combined with sedation could shorten the recovery time of patients [MD = −3.87, 95% CI (−5.43, −2.31), *P* < 0.001]. Due to high heterogeneity (*I*^2^ = 96.5%), a subgroup analysis was conducted according to the type of gastrointestinal endoscopy (gastroscopy/colonoscopy) and the number of kinds of sedatives used (Figure 7). The difference in recovery time between the intervention group and the control group was statistically significant in the subgroup concerning propofol used together with other sedative (s) in colonoscopy including only one study (13) [MD = −2.80, 95% CI (−3.05, −2.55), *P* < 0.001], in the subgroup of propofol alone in colonoscopy including two studies (16, 17) [MD = −4.78, 95% CI (−7.58, −1.99), *P* < 0.001], and in the subgroup of propofol alone in gastroscopy containing one study (20) [MD = −3.16, 95% CI (−3.94, −2.38), *P* < 0.001] as well. Sensitivity analysis showed that the results were stable (Figure 8). Egger's test indicated that there was no significant publication bias (*P* = 0.403 > 0.05) (Figure 9).

#### 3.4.4. The incidence of adverse events

The incidence of adverse events was recorded in nine articles (9, 13, 15–21). The adverse events were respiratory depression/bradycardia/hypotension/hypoxemia/nausea or vomiting/abdominal distension/dizziness/coughing/restlessness during or after gastrointestinal endoscopy. The incidence of all kinds of adverse events except abdominal distension in acupuncture combined with sedation was lower than in the control group. Nevertheless, the difference was only statistically significant in the incidence of hypotension, nausea and vomiting, and coughing between the two groups (*P* < 0.05) (Table 4), suggesting the potential of acupuncture specific to reducing the incidence of adverse events, including hypotension, nausea and vomiting, and coughing. As for acupuncture-related adverse events, merely two cases of acupuncture syncope were reported in a single study by Luo et al. (16).

**Table 4:** Meta-analysis of the incidence of adverse events.

**Adverse events**	**Number of studies**	**Intervention group**		**Control group**		***I* ^2^**	**RR (95% CI)**	***P***
		**Cases**	**Sample size**	**Cases**	**Sample size**			
Respiratory depression	2	5	100	13	100	0.0%	0.39 (0.14, 1.04)	0.061
Bradycardia	2	3	100	14	100	44.7%	0.25 (0.04, 1.78)	0.167
Hypotension	5	11	310	32	311	0.0%	0.40 (0.21, 0.77)	0.006^*^
Hypoxemia	3	16	190	27	191	42.3%	0.51 (0.18, 1.48)	0.219
Dizziness	1	0	60	2	60	0.0%	0.20 (0.01, 4.08)	0.296
Nausea or vomiting	7	15	500	53	501	0.0%	0.30 (0.17, 0.52)	0.000^*^
Abdominal distension	2	11	150	26	151	75.3%	1.05 (0.05, 20.66)	0.974
Coughing	2	5	190	21	190	0.0%	0.24 (0.09, 0.62)	0.003^*^
Restlessness	2	3	190	10	190	0.0%	0.30 (0.08, 1.08)	0.066

RR, risk ratio; CI, confidence interval; ^*^P < 0.05.

## 4. Discussion

### 4.1. Efficacy and safety of acupuncture combined with sedation in gastrointestinal endoscopy

As for the efficacy of acupuncture combined with sedation in gastrointestinal endoscopy, the results of the meta-analysis suggested that acupuncture combined with sedation reduced sedative consumption and wake-up time. However, as blinding was hardly possible due to the absence of sham/placebo acupuncture in the control groups of the included studies, a placebo effect could not be completely ruled out. Furthermore, due to the significant heterogeneity regarding these outcomes, additional studies are needed to reach definite conclusions.

The overall incidence of adverse effects was lower in the group of acupuncture combined with sedation than in the control group, indicating the efficacy of acupuncture in improving the safety of sedated gastrointestinal endoscopy. Only the incidence of hypotension, nausea and vomiting, and cough significantly differed between the intervention and control groups (*P* < 0.05). The more serious adverse effects, such as hypoxemia, bradycardia, and hypotension, which occurred more frequently, were all probably related to the sedative propofol. In addition, studies have reported that acupuncture combined with sedation was effective in suppressing stress responses and stabilizing hemodynamics in patients (9, 16, 18), thus less affecting vital signs than using sedatives alone.

The two cases of acupuncture syncope reported by Luo et al. (16) seem to suggest that acupuncture-related adverse events are rare and minor. However, such acupuncture-related adverse events were not followed up. Besides, underreporting of less significant adverse events in the clinical practice of acupuncture is also an indispensable factor (23). Therefore, further attention should be paid to monitoring the safety of acupuncture when it is combined with sedation in gastrointestinal endoscopy the future clinical research.

Therefore, a conclusion could be drawn with caution that the application of acupuncture in sedation in gastrointestinal endoscopy was considered effective. Nevertheless, the safety evaluation of acupuncture lacks sufficient evidence. The reduction of both wake-up time and the incidence of adverse events in the intervention group might be attributed to the lower amount of propofol used, considering the potential disadvantages of this sedative-hypnotic drug including rapid depression of consciousness and cardiovascular functions (3).

### 4.2. Evaluation of cognitive function

The choice of sedative for gastrointestinal endoscopy should consider the appropriate level of sedation, less hemodynamic influence, minimum postoperative cognitive dysfunction of patients, and early return to daily life (24). Despite limited sedative consumption in painless gastrointestinal endoscopy, patients may still experience transient cognitive impairment in memory, attention, and executive function during the recovery period from the sequelae of sedatives, hindering fine and complex daily activities shortly after discharge from the hospital (5, 25). Our meta-analysis result based on available studies showed that acupuncture significantly reduced patients' postoperative wake-up time in sedated gastrointestinal endoscopy. However, more attention should be paid to the recovery of cognitive functions after the examination, since no indicator concerning cognitive function is reported in the included studies.

For patients, the return to daily life after gastrointestinal endoscopy hinges on the timely recovery of cognitive function after sedation (25). As a rapid and reliable screening assessment tool for cognitive function, which is sensitive and valid in various cognition-affecting disorders, CogState computerized cognitive tests consisting of eight independent tasks and its simplified versions are often applied to the assessment of cognitive function (5, 25, 26). Tian et al. (5) utilized the CogState brief computerized test battery consisting of four selected tasks to measure postoperative cognitive function in patients undergoing colonoscopy with propofol sedation and found that the patients' psychomotor function and attention at discharge were impaired compared with before the examination, with their visual memory and working memory not significantly affected.

A study has recommended that patients receiving ambulatory surgery under any anesthesia should not drive motor vehicles within 24 h because of higher risks of traffic accidents (27). The updated version of Practice guidelines for postanesthetic care issued by the American Society of Anesthesiologists Task Force also recommended that the discharge of postanesthetic patients should be accompanied by a responsible adult (28). Therefore, the psychomotor function is often of substantial concern when studying patients' postoperative cognitive function. Tests such as Choice Reaction Time (CRT) (29), Number Connection Test (NCT) (30, 31), driving simulation test (30, 31), Digit Symbol Substitution Test (DSST) (32, 33), Modified Post Anesthesia Discharge Scoring (MPADS) system (34), and Trieger Dot Test (TDT) (32) have been used in different studies to investigate patients' postoperative psychomotor recoveries. Theodorou et al. (35) used a psychomotor test consisting of nine tests to evaluate more comprehensively. Considerably more work will need to be done using the scales above to assess postoperative cognitive function recovery of acupuncture combined with sedation, especially psychomotor function.

Furthermore, in relevant studies of propofol administration in gastrointestinal endoscopy, postoperative assessment of patients' cognitive function was often performed in the short term ranging from within 2 h after the examination (25) to before discharge from the hospital (5, 36), but a long-term post-discharge follow up is absent. Further studies, which combine short-term assessment with long-term follow-up, will need to be undertaken when exploring the effects of acupuncture combined with sedation on cognitive function.

### 4.3. Prospects

A previous systematic review published in 2004 by Lee and Ernst (12) compared the effectiveness of acupuncture, conventional sedation, and sham acupuncture in gastrointestinal endoscopy included only six clinical trials with problems such as small sample size, high heterogeneity, lack of unified outcome, and unclear provisions for primary outcomes, making a meta-analytical approach impossible. A meta-analysis recently published by Gao et al. (11) further found that in unsedated upper gastrointestinal endoscopy, acupuncture in addition to topical pharyngeal anesthesia (TPA) with lidocaine hydrochloride might better alleviate patients' discomfort during the examination compared with TPA alone. Wang et al. (10) compared the effects of acupuncture on colonoscopy through meta-analysis and found that acupuncture could significantly reduce the incidence of adverse events, shorten insertion time, relieve patients' pain, and improve patients' satisfaction, but no significant difference was found in propofol dosage between the intervention group and the control group according to the four RCTs included for their analysis. Our meta-analysis preliminarily suggested that acupuncture combined with sedation reduced sedative consumption and wake-up time.

Present studies have demonstrated a lack of application of placebo acupuncture or sham acupuncture in the control group (37–40). In order to minimize possible placebo effects, reduce the risk of bias, and improve both the methodological quality of the studies and the reliability of the results, the application of non-invasive sham/placebo acupuncture in the control group of future studies is recommended. Furthermore, the existing studies fail to provide data on endoscopic procedures other than colonoscopy and gastroscopy, such as endoscopic ultrasound (EUS) or endoscopic retrograde cholangiopancreatography (ERCP), where deep sedation with propofol is more necessary (41, 42). More detailed documentation of acupuncture-related adverse effects is required. The exact source of the heterogeneity in multiple outcome measures remained unclear, one possible explanation being the inconsistency of interventions across studies, which suggests the importance of the standardization of both acupuncture and sedation measures. Chen et al. (22) designed an orthogonal trial method to determine the optimized scheme of acupuncture combined with sedation for painless colonoscopy in different age groups, providing a possible solution for the problem. Anyway, more standardized, high-quality, multicenter, and large-scale RCTs with placebo acupuncture or sham acupuncture combined with sedation as the control group are needed, with standardization of therapeutic parameters such as acupoints, stimulation intensity and frequency, stimulation time of acupuncture, and sedative selection, in an attempt to better guide and promote the application of acupuncture combined with sedation in gastrointestinal endoscopy.

## 5. Conclusion

In view of all that has been mentioned so far, it may be supposed that acupuncture combined with sedation reduced sedative consumption and wake-up time compared with simple sedation in gastrointestinal endoscopy, which allows patients to regain consciousness more quickly after examination and overall reduces the risk of adverse effects. However, with the limited quantity and quality of relevant clinical studies, caution must be applied until more high-quality clinical studies verify and refine the conclusions.

## Data availability statement

The original contributions presented in the study are included in the article/supplementary material, further inquiries can be directed to the corresponding authors.

## Author contributions

YY: conception and design, analysis and interpretation of the data, drafting of the article. HJ, YL, and JL: analysis and interpretation of the data, critical revision of the article for important intellectual content. JH, GY, and XK: critical revision of the article for important intellectual content. XM: conception and design, critical revision of the article for important intellectual content. All authors contributed to the article and approved the submitted version.
